# Disparities in the participation and adherence of older adults in lifestyle-based multidomain dementia prevention and the motivational role of perceived disease risk and intervention benefits: an observational ancillary study to a randomised controlled trial

**DOI:** 10.1186/s13195-021-00904-6

**Published:** 2021-09-24

**Authors:** Nicola Coley, Delphine Coniasse-Brioude, Valérie Igier, Tristan Fournier, Jean-Pierre Poulain, Sandrine Andrieu, for the ACCEPT study group, Sandrine Andrieu, Nicola Coley, Virginie Gardette, Alain Grand, Valérie Igier, Delphine Coniasse Brioude, Maria Teresa Munoz Sastre, Jean Pierre Poulain, Tristan Fournier, Christelle Arandjelovic, Bruno Vellas, Stephane Oustric, Julie Subra

**Affiliations:** 1 Center for Epidemiology and Research in Population health (CERPOP), University of Toulouse, INSERM, UMR1295, 37 allées Jules Guesde, 31000 Toulouse, France; 2 Department of Epidemiology and Public Health, Toulouse University Hospital, Toulouse, France; 3 EA 7411- CERPPS, Université de Toulouse Jean Jaurès, Toulouse, France; 4 CNRS-Iris, Paris, France; 5 CERTOP UMR-CNRS 5044, Université de Toulouse Jean Jaurès, Toulouse, France

**Keywords:** Prevention, Lifestyle, Multidomain, Participation, Engagement, Adherence, Intervention, Population bias, Disparities

## Abstract

**Background:**

Preventive interventions for dementia are urgently needed and must be tested in randomised controlled trials (RCTs). Selection (volunteer) bias may limit efficacy, particularly in trials testing multidomain interventions and may also be indicative of disparities in intervention uptake in real-world settings. We identified factors associated with participation and adherence in a 3-year RCT of multidomain lifestyle intervention and/or omega-3 supplementation for prevention of cognitive decline and explored reasons for (non-) participation.

**Methods:**

Ancillary study during recruitment and follow-up of the 3-year Multidomain Alzheimer Preventive Trial (MAPT) conducted in in 13 memory centres in France and Monaco, involving 1630 community-dwelling dementia-free individuals aged ≥ 70 who were pre-screened for MAPT (1270 participated in MAPT; 360 declined to participate).

**Results:**

Response rates were 76% amongst MAPT participants and 53% amongst non-participants. Older individuals (odds ratio 0.94 [95% confidence interval 0.91–0.98] and those with higher anxiety (0.61 [0.47–0.79]) were less likely to participate in the trial. Those with higher income (4.42 [2.12–9.19]) and family history (1.60 [1.10–2.32]) or greater fear (1.73 [1.30–2.29]) of dementia were more likely to participate, as were those recruited via an intermediary (e.g. pension funds, local Alzheimer’s associations, University of the 3rd Age, sports clubs) (2.15 [1.45–3.20]). MAPT participants living in larger towns (0.71 [0.55–0.92]) and with higher depressive symptoms (0.94 [0.90–0.99]) were less likely to adhere to the interventions. Greater perceived social support (1.21 [1.03–1.43]) and cognitive function (1.37 [1.13–1.67]) predicted better adherence. Descriptively, the most frequent reasons for accepting and refusing to participate were, respectively, altruism and logistical constraints, but underlying motivations mainly related to (lack of) perceived benefits.

**Conclusions:**

Disparities in uptake of health interventions persist in older age. Those most at risk of dementia may not participate in or adhere to preventive interventions. Barriers to implementing lifestyle changes for dementia prevention include lack of knowledge about potential benefits, lack of support networks, and (perceived) financial costs.

**Trial registration:**

NCT00672685 (ClinicalTrials.gov)

**Supplementary Information:**

The online version contains supplementary material available at 10.1186/s13195-021-00904-6.

## Background

Given the growing burden of Alzheimer’s and other dementias [1], and a lack of effective treatment options, preventive interventions are urgently needed. Since modifiable lifestyle factors could account for up to 40% of dementia cases [2], attention has turned to non-pharmacological preventive interventions [3]. Several trials have shown promising results with this kind of intervention, echoing the results of observational studies, but there have also been many negative trials [3]. Currently, there is particular interest in multidomain interventions, simultaneously targeting multiple risk factors and/or behaviours, and numerous trials are being set up across the world [4], to confirm the promising results, in the primary analysis or certain at-risk subgroups, of the first multidomain trials [5–7].

One factor which could influence intervention efficacy in prevention trials is selection (volunteer) bias. Dementia prevention trial participants tend to be more educated and healthier than the general population of older adults [8], leading to a lower risk of dementia, and therefore potentially limited benefits from preventive interventions. Selection bias may be further emphasised during follow-up if subjects who drop out or who are non-adherent systematically differ from trial completers and adherent subjects. These selection biases could be even more pronounced in non-pharmacological trials encouraging behaviour modifications, particularly those using a multidomain approach and long intervention periods.

However, relatively little is known about participation and adherence in dementia prevention trials [8–13]. In particular, at the time of recruitment, dementia prevention trial participants have not yet been compared with individuals who were eligible but did not participate, and only a limited amount of purely descriptive data is available concerning reasons for participation, and occasionally non-participation, in such trials [12]. Furthermore, there has been no thorough assessment of the link between psychosocial characteristics and participation or adherence, and existing research has not been guided by a theoretical framework.

The Health Belief Model (HBM) was developed to explain and predict healthy or risky behaviours on the basis of individuals’ evaluations, perceptions, and beliefs [14]. According to this model, the probability of adopting preventive behaviours is determined by (i) sociodemographics, and (ii) psychosocial characteristics, such as personality (e.g. emotional stability), social support, perceived risk and control, and motivations, which give rise to five key perceptions and beliefs: perceived susceptibility and severity of illness (i.e. perceived threat), perceived benefits of behaviour and perceived barriers (i.e. ‘costs’ or personal investment) to behaviour, and general attitude towards health. Finally, there may be ‘cues to action’ (e.g. symptoms such as subjective memory problems, or family history of dementia) that influence readiness to engage in healthy behaviours.

Using the HBM as the main theoretical framework, supplemented with additional social constructs identified in our initial qualitative work [15], the aims of this multidisciplinary study, combining perspectives from epidemiology, public health, geriatrics, psychology and sociology, were to (i) identify socio-demographic, memory, and psychosocial factors associated with participation in a 3-year trial of a multidomain lifestyle intervention, and/or omega-3 supplementation, for the prevention of cognitive decline; (ii) explore reasons for participation and non-participation using factorial techniques in order to identify the main factors (dimensions) associated with (non-) participation; and (iii) amongst trial participants, to identify socio-demographic, psychosocial, and clinical factors associated with adherence.

## Methods

### Setting and participants

ACCEPT was a mixed-methods ancillary study conducted during recruitment and follow-up of the MAPT trial. This manuscript presents findings from the quantitative part of the ACCEPT study [15].

MAPT was a randomised controlled trial of a multidomain lifestyle intervention (cognitive training, physical activity, nutritional advice), omega-3 supplementation, or both interventions combined, versus placebo, for the prevention of cognitive decline in 1679 community-dwelling dementia-free subjects aged 70 and older with subjective memory problems, and/or a limitation in one instrumental activity of daily living, and/or slow walking speed. Full details have been published previously [5]. Subjects were recruited between 2008 and 2011 in 13 memory centres in France and Monaco, primarily via TV/radio/newspaper campaigns, memory centre consultations, and conferences or communications organised in conjunction with pension funds or senior organisations. Only limited details about the trial were given during the initial recruitment stage, and potential participants were pre-screened, either via telephone or in person, to ensure they met at least one of the three main pre-selection criteria. Those who were eligible based on these criteria were given full details about the trial and invited to participate and attend a full screening visit. At this time, regardless of whether they accepted or declined to participate in the MAPT trial, eligible subjects were also asked to fill in a questionnaire for the ACCEPT study and to return it using a pre-paid envelope.

### Protocol approvals, registrations, and patient consents

The MAPT trial protocol, including the ACCEPT sub-study, was approved by the Toulouse ethics committee (CPP SOOM II) and the French Health Authority, and trial participants provided written informed consent. The trial was registered on clinicaltrials.gov: NCT00672685.

### Data collection

We designed a multidimensional self-completion questionnaire (piloted with a group of 60 older individuals) for this study. There were two versions: one for individuals who agreed to participate in the MAPT trial, and one for those who declined. Both versions contained identical questions about socio-demographics, personal and family history of memory problems/AD, and psychosocial elements: perceived social support (4 items), emotional stability (6 anxiety items from the NEO Personality Inventory-Revised), health locus of control (18 items), and perceived risk of memory disorders/AD (4 items developed for this questionnaire, based on the literature). References used to construct the different elements of the questionnaire can be found in Additional file 1: Appendix 1.

Reasons for participation or non-participation (depending on whether or not the respondent had agreed to participate in the MAPT trial) were also explored using items based on the literature (see Additional file 1: Appendix 1 for references), eight individual semi-structured interviews conducted with older people, half of whom were participating in a dementia prevention trial at the time, and a focus group. Responses to the psychosocial questions and reasons for participation/non-participation were given on a 4-point Likert scale (ranging from ‘do not agree at all’ to ‘completely agree’).

For individuals who participated in the MAPT trial, clinical data concerning cognitive function, APOE genotype, physical and functional status, and cardiovascular risk factors were also collected, as described previously [5].

### Outcomes

Our primary outcome was participation in the MAPT trial, amongst eligible subjects. The secondary outcome was adherence, defined as the proportion of participants completing ≥ 75% of their assigned interventions (i.e. taking at least 75% of placebo/omega-3 capsules (verified by pill count), and, in the groups receiving the multidomain intervention, also attending at least 75% of the multidomain intervention sessions), amongst MAPT participants. Adherence was calculated over the entire 3-year follow-up period, regardless of dropout status, except for participants who died or dropped out due to medical reasons, for whom the adherence rate was calculated only until the time of dropout.

### Data analysis

Baseline characteristics of participants versus non-participants and adherent versus non-adherent participants were compared using *t* tests or Wilcoxon rank-sum tests for continuous variables and chi-square tests for categorical variables.

Reasons for (not) participating in the MAPT trial were first analysed descriptively, and then further explored using factor analyses (principal components) conducted on the raw questionnaire data. The number of factors was determined using the Scree test, and retained factor solutions were subjected to VARIMAX rotation (to identify independent factors). Items were retained only if they loaded strongly (> .5) on a single factor, with cross-loadings < .2.

Baseline factors associated with participation and adherence were analysed using multivariable multilevel logistic regression models. For each outcome (participation, adherence), bivariate models were run for all available candidate predictor variables, and those with a *p* value < 0.20 were included in multivariable analyses using a two-step process. First, separate multivariable models were run for socio-demographic, psychosocial/subjective variables, and (for the adherence analysis only) clinical variables. Then, variables that remained significantly (*p* < 0.05) associated with the outcome of interest, after a manual backwards stepwise selection procedure (i.e. sequentially removing variables with a *p* value > 0.05 from the model, starting with the variable with the highest *p* value, and verifying that there was no substantial impact on other variables in the model), were included together in a final multivariable model which was again subjected to a manual backwards stepwise selection procedure.

Analyses were performed using Stata version 14.1 (StataCorp LP, College Station, Texas) and Statistica version 13.2.

## Results

**Fig. 1:**
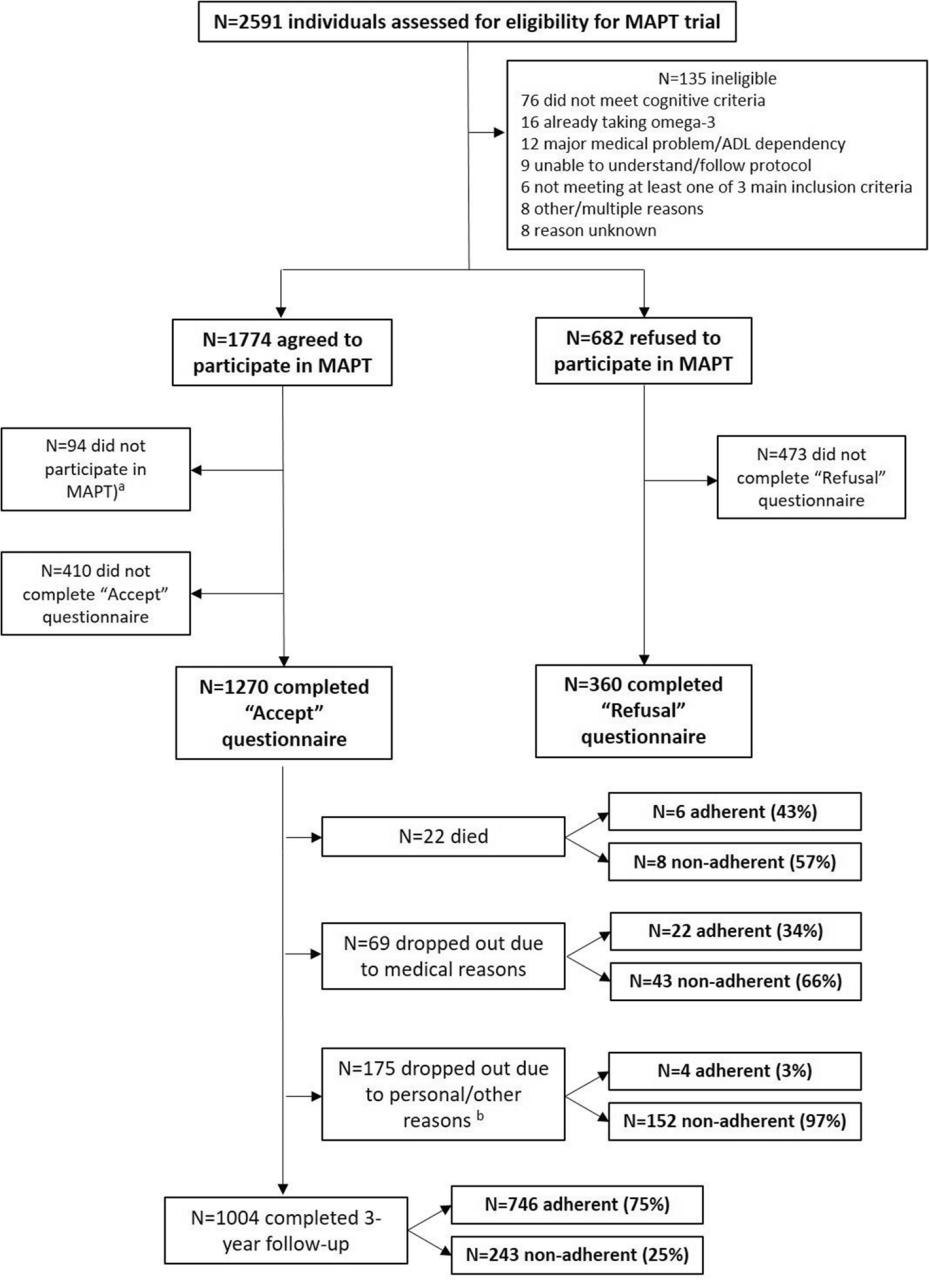
Flowchart. Adherence was defined as the proportion of participants completing ≥ 75% of their assigned interventions (calculated over the entire 3-year follow-up period, regardless of dropout status, except for participants who died or dropped out due to medical reasons, for whom the adherence rate was calculated only until the time of dropout). Adherence could not be calculated for some participants due to missing data (primarily regarding adherence to the omega-3 supplement/placebo). Superscript lowercase letter ‘a’ indicates inclusion visit not planned or not attended; superscript lowercase letter ‘b’ indicates including 8 subjects who did not attend the 3-year visit but who may have continued into the extended follow-up period

In total, 2591 individuals were assessed for eligibility for the MAPT trial during pre-screening, of whom 135 were ineligible. Of the remaining 2456, 1774 initially agreed to participate in the trial and 1680 were included (Fig. 1). Of these, 1270 (76%) completed an ACCEPT questionnaire (‘Accept’ version). Of the 682 eligible individuals who refused to participate in the trial after pre-screening, 360 (53%) completed an ACCEPT questionnaire (‘Refusal’ version).

Compared to participants, non-participants were older, more predominantly female and single, had a lower level of education and income, were more likely to have heard about the trial from a doctor, were less likely to have a family history of AD or related diseases, were less likely to consider memory problems to be a risk factor, had a lower perceived risk of AD, and were more anxious (Table 1).

**Table 1:** Baseline characteristics of non-participants and participants of the MAPT trial

	Non-participants (***N*** = 360)	Participants			***p***	
		All (***N*** = 1270)	Non-adherent (***N*** = 446)	Adherent (***N*** = 778)	Non-part. vs. part.	Adh. vs. non-adh.
**Age** (y), median [IQR]	76 [72–80]	75 [72–78]	75 [72–78]	74 [72–78]	**< 0.001**	**0.033**
**Female**, *N* (%)	252 (70.8)	810 (63.8)	273 (61.2)	508 (65.3)	**0.014**	0.152
**Level of education**,^a^*N* (%)					**< 0.001**	0.540
Low	99 (28.5)	247 (19.8)	93 (21.3)	144 (18.8)		
Intermediate	126 (36.3)	426 (34.1)	143 (32.7)	266 (34.6)		
High	122 (35.2)	577 (46.2)	201 (46.0)	358 (46.6)		
**Household monthly income**, *N* (%)					**< 0.001**	0.108
< 1000€	44 (14.3)	79 (6.7)	36 (9.0)	38 (5.2)		
1000–1999€	116 (37.7)	405 (34.6)	140 (35.0)	252 (34.4)		
2000–2999€	84 (27.3)	346 (29.5)	107 (26.8)	226 (30.8)		
3000–3999€	40 (13.0)	202 (17.3)	67 (16.8)	131 (17.9)		
> 4000€	24 (7.8)	139 (11.9)	50 (12.5)	86 (11.7)		
**Marital status**, *N* (%)					**0.034**	0.567
Single	35 (10.0)	72 (5.7)	21 (4.8)	46 (6.0)		
Married^b^	179 (51.1)	703 (56.0)	242 (54.9)	439 (57.0)		
Widowed	92 (26.3)	325 (25.9)	118 (26.8)	195 (25.3)		
Separated	44 (12.6)	156 (12.4)	60 (13.6)	90 (11.7)		
**Living alone**, *N* (%)	149 (42.8)	495 (39.6)	176 (40.3)	301 (39.2)	0.284	0.712
**Current working status**, *N* (%)					0.367	0.615
Full/part-time paid work	6 (1.7)	37 (2.9)	12 (2.8)	24 (3.1)		
Retired	323 (92.8)	1164 (92.5)	409 (93.6)	714 (92.1)		
Never worked	19 (5.5)	57 (4.5)	16 (3.7)	37 (4.8)		
**Town popn. size** > 200 000, *N* (%)	243 (67.5)	793 (62.4)	297 (66.6)	465 (59.8)	0.078	**0.018**
**First source of information about trial**, *N* (%)					**< 0.001**	**0.018**
Doctor	157 (44.7)	349 (27.7)	135 (30.8)	199 (25.7)		
Media	72 (20.5)	437 (34.7)	132 (30.1)	293 (37.9)		
Intermediary ^c^	122 (34.8)	472 (37.5)	172 (39.2)	282 (36.4)		
**Family history of AD or related diseases**,^**d**^*N* (%)					**0.006**	0.872
No	213 (64.5)	691 (56.7)	237 (55.9)	429 (57.3)		
Yes	75 (22.7)	378 (31.7)	136 (32.1)	236 (31.5)		
Do not know	42 (12.7)	141 (11.6)	51 (12.0)	84 (11.2)		
**Subjective memory complaint**, *N* (%)					0.232	0.565
No	68 (20.2)	203 (16.7)	77 (18.1)	122 (16.2)		
Yes	236 (70.0)	909 (74.6)	309 (72.7)	568 (75.5)		
Do not know	33 (9.8)	107 (8.8)	39 (9.2)	62 (8.2)		
**Reporting of memory complaint**, *N* (%)					0.054	0.719
Only to doctor	61 (17.9)	198 (16.0)	72 (16.8)	118 (15.5)		
Only to friends/family	62 (18.2)	309 (25.0)	101 (23.6)	196 (25.7)		
To doctor and friends/family	82 (24.0)	297 (24.0)	98 (22.9)	185 (24.3)		
To no one/no memory complaint	136 (39.9)	432 (35.0)	157 (36.7)	264 (34.6)		
**Impact of memory problems on everyday life**, *N* (%)					0.245	0.057
It bothers me a lot	11 (3.2)	55 (4.5)	28 (6.5)	23 (3.0)		
It bothers me a little	122 (35.5)	430 (34.8)	137 (32.0)	271 (35.7)		
It does not really bother me	119 (34.6)	460 (37.3)	161 (37.6)	287 (37.8)		
It does not bother me at all	59 (17.2)	210 (17.0)	75 (17.5)	127 (16.7)		
I do not have any memory problems	33 (9.6)	79 (6.4)	27 (6.3)	52 (6.8)		
**Memory problem pointed out by friends/family**, *N* (%)					0.549	0.168
Yes, very often	14 (4.7)	70 (6.3)	32 (8.4)	36 (5.3)		
Yes, sometimes	115 (39)	395 (35.7)	131 (34.4)	245 (35.9)		
Yes, rarely	83 (28.1)	337 (30.5)	120 (31.5)	204 (29.9)		
No	83 (28.1)	304 (27.5)	98 (25.7)	198 (29.0)		
**Memory problems considered to be a risk**, *N* (%)					**0.008**	0.103
Yes, it is a very big risk for developing diseases	66 (20.1)	324 (26.6)	126 (30.1)	190 (25.1)		
Yes, it could be a risk	205 (62.3)	753 (61.8)	238 (56.8)	485 (64.2)		
No, it is not a major risk	48 (14.6)	114 (9.4)	44 (10.5)	64 (8.5)		
No, it is not a risk at all	10 (3.0)	28 (2.3)	11 (2.6)	17 (2.3)		
**Perceived risk of Alzheimer’s disease**,^e^ median [IQR]	3.0 [2.8–3.5]	3.3 [2.8–3.5]	3.3 [2.9–3.8]	3.3 [2.8–3.5]	**0.001**	**0.003**
**Perceived social support**,^e^ median [IQR]	3.5 [2.8–4.0]	3.3 [2.8–4.0]	3.3 [2.8–3.8]	3.3 [3.0–4.0]	0.332	**0.002**
**Emotional stability**,^e^ mean (SD)	2.5 (0.6)	2.4 (0.6)	2.5 (0.6)	2.4 (0.6)	**0.012**	**0.009**
**Internal locus of control**,^e^ mean (SD)	2.7 (0.5)	2.8 (0.4)	2.8 (0.4)	2.8 (0.4)	0.123	0.483
**External locus of control (chance)**,^e^ mean (SD)	2.4 (0.7)	2.4 (0.6)	2.4 (0.6)	2.4 (0.6)	0.585	0.136
**External locus of control (powerful others)**,^e^ mean (SD)	2.7 (0.6)	2.7 (0.5)	2.7 (0.5)	0.7 (0.5)	0.438	0.561
**CDR 0.5**, *N* (%)	*N/A*	499 (40.8)	191 (42.8)	308 (39.6)	*N/A*	0.275
**APO4 ɛ4**, *N* (%)	*N/A*	230 (23.6)	66 (22.8)	164 (23.9)	*N/A*	0.728
**Hypercholesterolemia**, *N* (%)	*N/A*	352 (29.4)	132 (30.1)	220 (29.0)	*N/A*	0.684
**≥ 1 IADL limitation**, *N* (%)	*N/A*	58 (4.9)	28 (6.5)	30 (4.0)	*N/A*	0.055
**≥ 1 Fried frailty criteria**, *N* (%)	*N/A*	510 (43.7)	208 (48.6)	302 (40.8)	*N/A*	**0.010**
**CAIDE dementia risk score ≥ 6**, *N* (%)	*N/A*	1026 (86.1)	378 (87.5)	648 (85.3)	*N/A*	0.284
**BMI**, *N* (%)	*N/A*				*N/A*	**0.041**
18.5–24.9		522 (43.2)	169 (38.6)	353 (45.8)		
25–29.9		503 (41.6)	193 (44.1)	310 (40.2)		
≥ 30		184 (15.2)	76 (17.4)	108 (14.0)		
**SBP**, median [IQR]	*N/A*	140 [130–152]	140 [130–153]	140 [130–151]	*N/A*	0.492
**DBP**, mean (SD)	*N/A*	79.3 (11.1)	79.3 (11.3)	79.3 (11.1)	*N/A*	0.986
**Cognitive composite score**, mean (SD)	*N/A*	0.07 (0.65)	− 0.04 (0.69)	0.13 (0.61)	*N/A*	**< 0.001**
**Depressive symptoms (GDS score)**, median [IQR]	*N/A*	3 [1–4]	3 [2–5]	2 [1–4]	*N/A*	**< 0.001**
**Subjective memory function**, mean (SD)	*N/A*	49.8 (16.8)	50.8 (17.2)	49.3 (16.5)	*N/A*	0.130

^a^ Low education = primary school certificate or lower; intermediate education = middle/vocational school; high education = high school diploma (e.g. baccalaureate) or higher; ^b^ or living as a couple; ^c^ conferences organized by pension fund organisations, word of mouth, participants from previous studies, and via organisations such as local Alzheimer’s associations, University of the 3rd Age, sports clubs and home-help organisations; ^d^ blood relative with memory problems, AD or ‘senility’; ^e^ score/4; higher scores indicate, respectively: greater perceived risk of Alzheimer’s disease, more social support, less emotional stability (i.e. more anxious), higher importance to internal locus of control, higher importance to external locus of control/chance, higher importance to external locus of control/medical professionals

### Reasons for participation

**Fig. 2:**
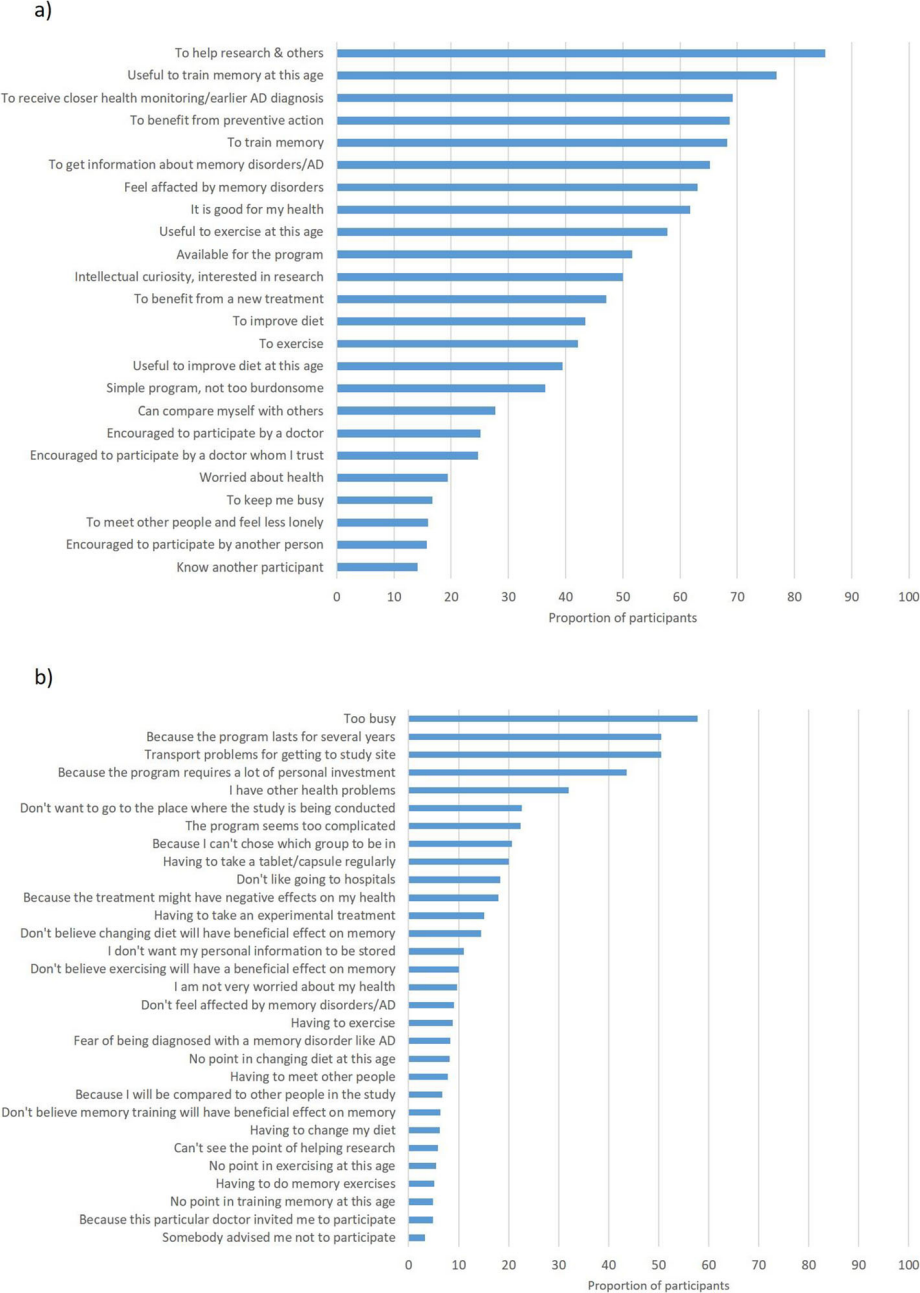
Reasons for accepting and refusing to participate in the MAPT trial. **a** Reasons for accepting: percentage of participants who declared that they completely agreed that this was a reason for participating in the MAPT trial (*N* = 1251). **b** Reasons for refusing: percentage of non-participants who declared that they completely agreed that this was a reason for not participating in the MAPT trial (*N* = 317)

Figure 2a shows the proportion of participants who completely agreed that the indicated reason was a reason why they decided to participate in the trial (descriptive analysis). The most frequently cited reasons were as follows: wanting to help research and other people (85% of participants), believing that memory training could be useful at their age (77%), wanting to receive closer health monitoring and/or an earlier AD diagnosis (69%), wanting to benefit from a preventive action (69%), and wanting to do memory training (68%).

The factor analysis indicated there were three underlying factors explaining 41% of the total variance amongst the reasons for participation (Table 2; Additional file 1: Appendix 2). The first factor, explaining 20% of the variance, related to ‘perceived benefits’, the second, explaining 12% of the variance, related to ‘social influences’, and the third, explaining 10% of the variance, related to ‘altruism and availability’. Measures of internal consistency (Cronbach’s alpha) were acceptable for factors 1 and 2 (0.86 and 0.75, respectively), but low for factor 3 (0.46).

**Table 2:** Exploratory factor solutions showing the underlying dimensions of reasons for (a) participating and (b) not participating

Items	Factor loadings			Items	Factor loadings		
	1	2	3		1	2	3
(a) Reasons for accepting to participate				(b) Reasons for refusing to participate			
***Factor 1: ‘Perceived benefits’***				***Factor 1: ‘Lack of interest’***			
To help me improve my diet	**.75**			I do not see the point of exercising at my age	**.78**		
To do physical activity	**.73**			I do not think that memory exercises will have a beneficial effect on my memory	**.67**		
To train my memory	**.72**			I do not see the point of training my memory at my age	**.66**		
To receive a preventive action	**.70**			I do not think that exercising will have a beneficial effect on my memory	**.64**		
I can see the point of changing my diet at this age	**.67**			I do not see the point of changing my diet at my age	**.63**		
To receive a new treatment	**.64**			I do not think that changing my diet will have beneficial effects on my memory	**.61**		
I can see the point of training my memory at this age	**.63**			I do not see the point of helping research	**.56**		
I can see the point of doing physical activity at this age	**.61**			I do not feel concerned by memory disorders such as Alzheimer’s disease	**.52**		
***Factor 2: ‘Social influences’***				***Factor 2: ‘Lack of control’***			
Because someone else (e.g. family member/friend) advised me to take part in the program		**.69**		Because the treatment could possibly have negative effects on my health		**.69**	
Because a doctor invited me to take part in the program		**.68**		Because the treatment to be taken is still under study		**.68**	
Because a doctor in whom I have confidence invited me to take part in the program		**.65**		Because I can be compared to other people in the study		**.67**	
Taking part in the program will help to keep me busy		**.61**		I am worried that I will find out I have a memory disorder like Alzheimer’s disease		**.64**	
To meet other people and feel less lonely		**.61**		I do not want my information to be held		**.60**	
				Because I cannot choose which group I will be in		**.59**	
***Factor 3: ‘Altruism and availability’***				***Factor 3: ‘Perceived constraints’***			
I have enough free time for this program			**.66**	Because the program lasts for several years			**.81**
In order to help move research forward and be useful to other people			**.65**	Because the program requires a lot of personal investment			**.80**
Because of intellectual curiosity and because I am interested in research			**.53**	I do not have enough time for the program			**.74**
**Eigen values**	**4.94**	**3**	**2.24**	**Eigen values**	**4.96**	**4.76**	**2.54**
**Percentage of variance**	**20**	**12**	**9**	**Percentage of variance**	**16**	**15**	**8**

Only items which loaded on a single factor (loading > .50, and cross-loadings < .2) are shown in this table. Factor loadings for the full set of items are shown in Additional file 1: Appendix 2

### Reasons for non-participation

Figure 2b shows the proportion of non-participants who completely agreed that the indicated reason was a reason why they decided to not to participate in the trial. The most frequently cited reasons were as follows: being too busy (58%), the fact that the program was due to last for several years (50%), and transport problems (50%).

The factor analysis indicated there were three underlying factors explaining 39% of the total variance amongst the reasons for non-participation (Table 2; Additional file 1: Appendix 2). The first factor, explaining 16% of the variance, was related to ‘lack of interest’, the second, explaining 15% of the variance, was related to ‘lack of control’, and the third, explaining 8% of the variance, was related to ‘perceived constraints’. Internal consistency was acceptable for all three factors (Cronbach’s alpha = 0.80–0.83–0.82).

### Factors associated with participation and adherence

In the final multivariable analysis (Table 3), individuals who were older or more anxious were less likely to participate in MAPT, and those with higher income, a family history, or greater fear of AD were more likely to participate. Additionally, compared to individuals who first heard about the trial through a doctor, those who first heard about the trial through the media or through an intermediary (e.g. organisations such as pension funds, local Alzheimer’s associations, University of the 3rd Age, and sports clubs), were more likely to participate.

**Table 3:** Factors associated with participation and adherence (final multivariable logistic regression models)

	Factors associated with participation (***N*** = 1267)			Factors associated with adherence (***N*** = 1155)		
	OR	95%CI	***p***	OR	95% CI	***p***
**Age** (years)	0.94	0.91, 0.98	**0.001**	**-**	**-**	**-**
**Household monthly income**			**< 0.001**	**-**	**-**	**-**
< 1000€	1.00					
1000–1999€	2.20	1.27, 3.80	**0.005**			
2000**–**2999€	2.56	1.47, 4.47	**0.001**			
3000**–**3999€	3.91	2.07, 7.38	**< 0.001**			
> 4000€	4.42	2.12, 9.19	**< 0.001**			
**Town popn. size** > 200 000	**-**	**-**	**-**	0.71	0.55, 0.92	**0.009**
**First source of information about trial**			**< 0.001**	**-**	**-**	**-**
Doctor	1.00					
Media	1.67	1.09, 2.57	**0.019**			
Intermediary^a^	2.15	1.45, 3.20	**< 0.001**			
**Family history of AD or related disorders**^b^			**0.036**	.	.	.
No	1.00					
Yes	1.60	1.10, 2.32	**0.013**			
Do not know	1.38	0.82, 2.24	0.228			
**Perceived risk of AD**^c^	1.73	1.30, 2.29	**< 0.001**	**-**	**-**	**-**
**Perceived social support**^c^	.	.	.	1.21	1.03, 1.43	**0.024**
**Emotional stability**^c^	0.61	0.47, 0.79	**< 0.001**	**-**	**-**	**-**
**BMI, kg/m**^**2**^	*N/A*	*N/A*	*N/A*			**0.040**
18.5**–**24.9				1.00		
25**–**29.9				0.75	0.57, 0.98	**0.035**
≥ 30				0.68	0.47, 0.97	**0.036**
**Cognitive function (composite*z*score)**	*N/A*	*N/A*	*N/A*	1.37	1.13, 1.67	**0.001**
**Depressive symptoms (GDS)**	*N/A*	*N/A*	*N/A*	0.94	0.90, 0.99	**0.024**

The table includes variables that remained significant in either the multivariate ‘participation’ or the multivariate ‘adherence’ model after the backwards stepwise selection procedures

‘-’ denotes variables that were included in the multivariable models, but did not remain in the final model.

Education, sex, marital status, internal locus of control, and belief that memory problems are a risk factor were additionally included in the multivariable participation models, but did not remain in the final model.

Sex, impact of memory problems on everyday life, belief that memory problems are a risk factor, emotional stability, external locus of control (chance), subjective memory function, altruisim as a reason for participating, functional status, and frailty were additionally included in the multivariable adherence models, but did not remain in the final model

‘.’ denotes variables that were not included in the multivariable models (*p* ≥ 0.20 in bivariate analysis).

‘N/A’ denotes variables that were not assessed as predictors of participation (as they were not available for non-participants)

*BMI*, Body mass index, *GDS*, Geriatric Depression Scale

^a^Conferences organized by pension fund organisations, word of mouth, participants from previous studies, and via organisations such as local Alzheimer’s associations, University of the 3rd Age, sports clubs and home-help organisations; ^b^ blood relative with memory problems, AD or ‘senility’; ^c^ score/4; higher scores indicate, respectively: greater perceived risk of Alzheimer’s disease, more social support, less emotional stability (i.e. more anxious)

In the adherence analysis, which additionally included clinical variables and reasons for participation as candidate predictor variables (see Table 1 for unadjusted analysis), five factors remained in the final multivariable model (Table 3). Participants living in larger towns and those with a higher depression score or higher BMI were less likely to adhere to their assigned interventions, whilst those with greater perceived social support or better cognitive function were more likely to adhere.

## Discussion

This in-depth study of participation of older adults in a dementia prevention trial presents two major findings. First, there were two levels of selection bias during the trial: individuals who agreed to participate differed from those who refused, and, amongst participants, those who adhered to the interventions differed from those who did not. This may limit the detection of potential intervention effects and the generalisability of results in trial settings and, on a public health level, likely reflects disparities in the uptake of preventive interventions in real-world settings. Second, although in the descriptive analyses, the most commonly cited reasons for accepting and refusing to participate were, respectively, altruism and logistical constraints, more in-depth analyses showed that the main underlying motivations concerning the decision to participate (or not) in a dementia prevention trial related to perceived benefits for participants, and lack of interest for non-participants.

Personal experience and perceptions of AD and related disorders were associated with participation in this dementia prevention trial, with individuals with greater fear of AD or those with a family history being more likely to take part. Although not previously studied in this context, family history and/or perceived threat of disease have been found to be associated with interest in receiving health education about chronic diseases [16], health protective behaviours [17], and willingness to participate in cardiovascular prevention trials [18]. Furthermore, prior contact with a person with dementia and identifying dementia as a health issue of personal concern were associated with taking actions to improve brain health [19]. Participation in our trial was related to a specific fear of AD/memory disorders, rather than anxiety in general, which was a barrier to participation. There were, however, no associations between participation and any of the other subjective or psycho-social variables, notably those relating to the health locus of control.

Although medical authority might influence older adults’ decisions concerning participation in prevention trials [20], in this trial, individuals recruited via a doctor were less likely to participate than those recruited via other sources. Although our analysis was adjusted for socio-demographic and other variables, we did not adjust for health status (since data were not available for the non-participants), meaning this result could be a reflection of underlying poorer health status amongst individuals recruited by a doctor. Indeed, a preliminary analysis suggested that MAPT participants recruited by a doctor were older and had poorer cognitive, physical, and functional status than those who were recruited via other methods (data not shown). Nonetheless, individuals who responded to media advertisements about the trial or attended conferences where the trial was presented, for example, had to be more proactive about participating, and probably only did so if they had a sufficiently high enough level of interest in participating in the first place. A better understanding of the characteristics of prevention trial participants recruited via different methods could provide insight into how best to reach specific subpopulations of individuals, which may be of particular use for more tailored approaches to dementia prevention.

As expected [8, 11, 13], younger age was strongly associated with participation in the trial, but, surprisingly [8, 13], education was not. However, the level of education was relatively high even amongst the non-participants, which may have limited its discriminatory effect in this population. Furthermore, education may have been a proxy for income in previous studies. There was a strong linear relationship between income and the odds of participating in our study, and perceived financial costs have previously been suggested to be a major barrier to access to preventive interventions, particularly those targeting lifestyle factors [21].

The factors associated with adherence were different to those associated with participation, perhaps because the influence of the latter was already taken into account at the initial participation level. Of note, participants with greater social support, which may protect against dementia [22], were more likely to adhere to the preventive interventions offered in the MAPT trial. Social support has also been identified as a facilitator to the uptake and maintenance of healthy behaviours in midlife [21]. Interestingly, although our measure of perceived social support (which assessed whether or not individuals had people around them who, when needed, could listen to/comfort them, look after them and provide material help, give advice, and provide reassurance) was associated with adherence, marital status and living alone were not. This suggests that the quality of social support may be more important in facilitating adherence, rather than simply the presence or quantity of social contacts.

Furthermore, participants living in smaller towns (population ≤ 200,000) were more adherent than those living in larger towns, perhaps because travel to the study centres was easier, and also because they may have had a greater sense of community spirit and greater attachment to the local doctors involved in the study.

We studied reasons for (non-) participation using an original approach, with the aim of identifying the underlying dimensions influencing the decision to participate (or not) in a multidomain dementia prevention trial. Of the three factors underlying the decision to participate, the first, ‘perceived benefits’ (e.g. being able to train one’s memory, or benefit from a new treatment), directly relates to the HBM and reflects the results of previous studies of older people’s willingness to participate in clinical trials [23, 24]. The second factor, ‘social influences’, demonstrates that the decision to participate is influenced by other people, such as family, friends, and medical professionals. The third factor related to ‘altruism and availability’, which are frequently cited motivators for participation in prevention trials [20, 23, 24]. Although altruism was the most frequently cited reason for participation in the descriptive analysis, the more in-depth analysis suggested that the notion of personal benefits may be an even more important consideration for older adults in the decision to participate in a prevention trial, which is similar to the results of our previous study of older Europeans’ motivations for participating in an eHealth prevention trial [20].

There were also three underlying factors influencing the decision not to participate. The first, ‘lack of interest’, showed that people refused to participate because they did not believe the interventions offered in the study were effective and/or necessary. Such beliefs could be related to their general attitude towards health, part of the HBM. Lack of interest was also a reason for non-participation in a previous dementia prevention trial [12]. Our results suggest that education and communication about dementia risk and the potential effects of lifestyle factors should be improved. Even though there has been substantial progress in the field of dementia prevention since our trial was conducted, it is likely that the advances in knowledge have not yet fully filtered through from the scientific community to the general public [25]. This may be, in part at least, due to difficulties in formulating public health messages about dementia prevention, given that levels of evidence, particularly in relation to interventions, remain sub-optimal for the most part and that the effects of certain risk factors may be limited to certain life periods, certain sub-populations, or certain types of dementia [26]. Furthermore, knowledge about risk and protective factors for Alzheimer’s disease is known to decrease with increasing age and decreasing level of education [27], suggesting that those with increased risk of dementia, who may be those likely to obtain the most benefit from interventions, may know the least about its risk factors.

The second factor influencing non-participation, ‘lack of control’, included items such as the fact that the treatment proposed was still under study and fear of being diagnosed with AD. Concerns about experimental treatments have previously been associated with refusal to participate in clinical trials [24], but to our knowledge, fear of being diagnosed with dementia has not previously been explored as a reason for non-participation in a prevention trial. The third factor directly relates to the HBM and shows that ‘perceived costs’, notably in terms of time investment, are an important consideration for potential prevention trial participants, as noted previously [11, 12, 28].

## Limitations

This study presents a number of strengths and original features, notably that it was a specifically designed sub-study of a dementia prevention trial, performed during an actual trial recruitment period, rather than assessing willingness to participate in a hypothetical trial, and we were able to question both participants and non-participants in a similar manner. Furthermore, reasons for participation and non-participation were examined using a multivariate/dimensional approach, rather than purely descriptively. However, the non-participants who responded to this study are probably not representative of all of the individuals who were eligible for the MAPT trial but did not participate, since we only studied those who had nonetheless accepted to go through the pre-screening procedure, and the response rate amongst these non-participants was only 53%. Nonetheless, we detected numerous differences between participants and non-participants, which would likely be further emphasised with a more representative sample of non-participants.

Barriers associated with participating in a trial of a preventive supplement may be different to those associated with participating in a preventive lifestyle intervention, with the latter kind of intervention requiring participants to be much more motivated, but we did not formally distinguish between the two kinds of barriers in our study. Nonetheless, in the same way that some factors predicting adherence are common to both lifestyle and supplement interventions [10], there may be some common motivators (for example, altruistic reasons, or having a strong belief in prevention) for participating in a prevention trial, regardless of the type of intervention.

We examined factors associated with both participation and adherence in the same sample using extensive multivariable analyses including psychosocial variables, notably those relating to fear and family history of AD. However, we did not have any objective health status variables for the non-participants, we did not measure self-efficacy, a key predictor of adherence [29], and we only assessed baseline predictors of adherence.

Finally, our study was multidisciplinary and based on a theoretical framework. However, although the HBM has been widely used in the literature, it remains nonetheless open to criticism, notably because it may not take into account all factors influencing preventive behaviours, particularly life events, ageing dynamics, and social interactions and circumstances [30–32].

## Conclusions and recommendations

Disparities in the uptake of health interventions seem to persist even in older age, and certain barriers may discourage those at greatest risk of dementia from accessing and/or adhering to preventive interventions. Further work is required to better identify and overcome these barriers, in order to increase participation and equality in uptake of dementia prevention strategies, both in research and public health settings, particularly given the recent publication of dementia prevention guidelines by the World Health Organization [33].

Our results suggest that education and communication about dementia risk and the potential effects of lifestyle factors, notably for those at highest risk of developing dementia, should be improved. It should also be underlined that healthy lifestyle changes can be made without incurring major financial costs. Furthermore, the importance of support networks, including family, friends, and medical professionals, in encouraging people to take action to improve their health should not be neglected. In terms of designing interventions, greater support and encouragement may need to be provided for older individuals, those with depression, anxiety, or limited social support, and those who are overweight or obese, perhaps through a more personalised approach to dementia prevention. Finally, in research settings, it will be important to reassure those with concerns about participating in research studies and also to emphasise the potential benefits of obtaining an earlier diagnosis of any potentially clinically significant memory problems, whilst acknowledging many older adults’ fear of dementia.

## Supplementary Information

**Additional file 1:Appendix 1.** References used to develop the ACCEPT study questionnaire. **Appendix 2.** Exploratory factor analysis of reasons for (a) participating and (b) not participating.

## Data Availability

The datasets used and/or analysed during the current study are available from the corresponding author on reasonable request.
