# Supplementary material for: Disparities in the participation and adherence of older adults in lifestyle-based multidomain dementia prevention and the motivational role of perceived disease risk and intervention benefits: an observational ancillary study to a randomised controlled trial
Source: Alzheimers Res Ther. 2021 Sep 24;13:157. doi: 10.1186/s13195-021-00904-6 (PMC8464095; doi:10.1186/s13195-021-00904-6)
Supplement: Supplementary file 1 — Additional file 1:Appendix 1. References used to develop the ACCEPT study questionnaire. Appendix 2. Exploratory factor analysis of reasons for (a) participating and (b) not participating. [file 13195_2021_904_MOESM1_ESM.docx]

**Appendix 1. References used to develop the ACCEPT study questionnaire**

Social support items:

- Rascle N, Bruchon-Schweitzer M, Sarason IG. Short form of Sarason's Social Support Questionnaire: French adaptation and validation. *Psychol Rep* 2005; **97**(1): 195-202.
- Sarason IG, Levine HM, Sarasib BR. Assessing social support : the social support questionnaire. *Journal of Personality and Social Psychology* 1983; **44**(1): 127-39.
- Bruchon- Schweitzer M. Psychologie de la Santé. Modèles, concepts et méthodes. Paris: Dunod; 2002.

Emotional stability items:

- Costa PT, McCrae RR. Revised NEO Personality Inventory (NEO- PI-R) and NEO Five Factor Inventory (NEO- FFI) professional manual. Odessa, FL, 1992.
- Rolland JP. Qualités psychométriques de la traduction française de l’inventaire de personnalité NEO PI R (étude préliminaire). *Revue Française et Francophone de Psychiatrie et de Psychologie Médicale* 1998; **18**: 139-44.

Health locus of control items:

- Wallston KA, Wallston BS, De Vellis RF. Development of the Multidimentional Health Locus of Control (MHLC) Scales. *Health Education Monographs* 1978; **6**(161-170).
- Wallston KA, Stein MJ, Smith CA. Form C of the MHLC scales: a condition-specific measure of locus of control. *J Pers Assess* 1994; **63**(3): 534-53.
- Bruchon- Schweitzer M. Psychologie de la Santé. Modèles, concepts et méthodes. Paris: Dunod; 2002.

Perceived risk of memory disorders/AD items:

- Roberts JS, Connell CM, Cisewski D, Hipps YG, Demissie S, Green RC. Differences between African Americans and whites in their perceptions of Alzheimer disease. *Alzheimer Dis Assoc Disord* 2003; **17**(1): 19-26.
- Hessel A, Gunzelmann T, Geyer M, Brahler E. [Utilization of medical services and medication intake of patients over 60 in Germany--health related, social structure related, socio-demographic and subjective factors]. *Z Gerontol Geriatr* 2000; **33**(4): 289-99.
- Nexoe J, Kragstrup J, Sogaard J. Decision on influenza vaccination among the elderly. A questionnaire study based on the Health Belief Model and the Multidimensional Locus of Control Theory. *Scand J Prim Health Care* 1999; **17**(2): 105-10.
- Reventlow S, Bang H. Brittle bones: ageing or threat of disease exploring women's cultural models of osteoporosis. *Scand J Public Health* 2006; **34**(3): 320-6.
- Tang CS, Wong CY. Psychosocial factors influencing the practice of preventive behaviors against the severe acute respiratory syndrome among older Chinese in Hong Kong. *J Aging Health* 2005; **17**(4): 490-506.

Reasons for participation/non-participation:

- Boles M, Getchell WS, Feldman G, McBride R, Hart RG. Primary prevention studies and the healthy elderly: evaluating barriers to recruitment. *J Community Health* 2000; **25**(4): 279-92.
- Fitzpatrick AL, Fried LP, Williamson J, et al. Recruitment of the elderly into a pharmacologic prevention trial: the Ginkgo Evaluation of Memory Study experience. *Contemp Clin Trials* 2006; **27**(6): 541-53.
- Lerman C, Rimer BK, Daly M, et al. Recruiting high risk women into a breast cancer health promotion trial. *Cancer Epidemiol Biomarkers Prev* 1994; **3**(3): 271-6.
- Rimer BK, Schildkraut JM, Lerman C, Lin TH, Audrain J. Participation in a women's breast cancer risk counseling trial. Who participates? Who declines? High Risk Breast Cancer Consortium. *Cancer* 1996; **77**(11): 2348-55.
- Sample DA, Sinicrope PS, Wargovich MJ, Sinicrope FA. Post-study aspirin intake and factors motivating participation in a colorectal cancer chemoprevention trial. *Cancer Epidemiol Biomarkers Prev* 2002; **11**(3): 281-5.
- Tolmie EP, Mungall MM, Louden G, Lindsay GM, Gaw A. Understanding why older people participate in clinical trials: the experience of the Scottish PROSPER participants. *Age Ageing* 2004; **33**(4): 374-8.

**Appendix 2. Exploratory factor analysis of reasons for (a) participating and (b) not participating**

| 1. **Reasons for Accepting to participate** | | | |  | 1. **Reasons for Refusing to participate** | | | |
| --- | --- | --- | --- | --- | --- | --- | --- | --- |
| **Items** | **Factor loadings** | | |  | **Items** | **Factor loadings** | | |
|  | **1** | **2** | **3** |  |  | **1** | **2** | **3** |
| To help me improve my diet | .75 |  |  |  | I don’t see the point of exercising at my age | .78 |  |  |
| To do physical activity | .73 |  |  |  | I don’t think that memory exercises will have a beneficial effect on my memory | .67 |  |  |
| To train my memory | .72 |  |  |  | I don’t see the point of training my memory at my age | .66 |  |  |
| To receive a preventive action | .70 |  |  |  | I don’t think that exercising will have a beneficial effect on my memory | .64 |  |  |
| I can see the point of changing my diet at this age | .67 |  |  |  | I don’t see the point of changing my diet at my age | .63 |  |  |
| To receive a new treatment | .64 |  |  |  | I don’t think that changing my diet will have beneficial effects on my memory | .61 |  |  |
| I can see the point of training my memory at this age | .63 |  |  |  | I don’t see the point of helping research | .56 |  |  |
| I can see the point of doing physical activity at this age | .61 |  |  |  | I do not feel concerned by memory disorders such as Alzheimer’s disease | .52 |  |  |
| I could be monitored and potentially receive an earlier diagnosis of a memory disorder | .53 | .04 | .35 |  | Because of having to exercise | .56 | .30 | .02 |
| I could get information about memory disorders like Alzheimer’s disease | .46 | .16 | .41 |  | Because of having to do memory training | .55 | .36 | -.09 |
| Because it’s good for my health | .39 | .32 | .04 |  | Because of having to change my diet | .54 | .21 | .19 |
| I am concerned about memory disorders such as Alzheimer’s disease | .37 | .01 | .21 |  | I am not worried about my health | .41 | .11 | .00 |
| Because someone else (e.g. family member/friend) advised me to take part in the program |  | .69 |  |  | Because the treatment could possibly have negative effects on my health |  | .69 |  |
| Because a doctor invited me to take part in the program |  | .68 |  |  | Because the treatment to be taken is still under study |  | .68 |  |
| Because a doctor in whom I have confidence invited me to take part in the program |  | .65 |  |  | Because I can be compared to other people in the study |  | .67 |  |
| Taking part in the program will help to keep me busy |  | .61 |  |  | I am worried that I will find out I have a memory disorder like Alzheimer’s disease |  | .64 |  |
| To meet other people and feel less lonely |  | .61 |  |  | I don’t want my information to be held |  | .60 |  |
| I know someone else who is taking part in this program or a similar program |  | .49 |  |  | Because I can’t choose which group I will be in |  | .59 |  |
| Because I will be compared to other people in the study | .18 | .37 | .32 |  | This program seems too complicated | .19 | .57 | .30 |
| I have enough free time for this program |  |  | .66 |  | Because I will have to meet other people | .32 | .48 | -.06 |
| In order to help move research forward and be useful to other people |  |  | .65 |  | Because I don’t like going to hospitals | .18 | .46 | .14 |
| Because of intellectual curiosity and because I am interested in research |  |  | .53 |  | Because it was this doctor who invited me to participate in the program | .23 | .45 | -.14 |
| I am worried about my health | .46 | .05 | .51 |  | I don’t want to go to the place where the study is being conducted | .17 | .44 | .25 |
| This program seems simple and not too burdensome | .18 | .31 | .35 |  | I have other health problems | .23 | .37 | .21 |
|  |  |  |  |  | Because someone else advised me not to participate in the program | .16 | .37 | -.02 |
|  |  |  |  |  | Because the program lasts for several years |  |  | .81 |
|  |  |  |  |  | Because the program requires a lot of personal investment |  |  | .80 |
|  |  |  |  |  | I don’t have enough time for the program |  |  | .74 |
|  |  |  |  |  | I am worried that I will have transport problems for getting to the study center | .02 | .23 | .32 |
| **Eigen values** | **4.94** | **3** | **2.24** |  | **Eigen values** | **4.96** | **4.76** | **2.54** |
| **Percentage of variance** | **20** | **12** | **9** |  | **Percentage of variance** | **16** | **15** | **8** |

Factor loadings >.50 are shown for items which loaded onto a single factor. For the other items, all factor loadings are shown.
